# Profile of the Main Representatives of Sphingolipid Metabolism in the Maxillary and Mandibular Periosteum of Patients with Dentofacial Deformities After Osteosynthesis Using Titanium Implants

**DOI:** 10.3390/jcm14061929

**Published:** 2025-03-13

**Authors:** Bożena Antonowicz, Agnieszka Błachnio-Zabielska, Urszula Chlabicz, Mateusz Maciejczyk, Jan Borys, Kamila Łukaszuk, Sara Zięba, Roberto Lo Giudice, Giuseppe Lo Giudice, Mariusz Szuta, Anna Zalewska

**Affiliations:** 1 Department of Dental Surgery, Medical University in Bialystok, 15-089 Białystok, Poland; 2 Department of Hygiene, Epidemiology and Metabolic Disorders, Medical University of Bialystok, 15-089 Białystok, Poland; agnieszka.blachnio-zabielska@umb.edu.pl (A.B.-Z.); urszula.chlabicz@umb.edu.pl (U.C.); 3 Department of Hygiene, Epidemiology and Ergonomics, Medical University of Bialystok, 15-089 Białystok, Poland; 4 Department of Maxillofacial and Plastic Surgery, Medical University of Bialystok, 15-089 Białystok, Poland; 5 Department of Restorative Dentistry, Medical University of Bialystok, 15-089 Białystok, Polandanna.zalewska1@wp.pl (A.Z.); 6 Department of Human Pathology of the Adult and Evolutive Age G. Barresi, Messina University, 98100 Messina, Italy; roberto.logiudice@unime.it; 7 Department of Biomedical and Dental Sciences and Morphofunctional Imaging, Messina University, 98100 Messina, Italy; logiudiceg@unime.it; 8 Department of Oral Surgery, Jagiellonian University Medical College, 31-008 Kraków, Poland; 9 Independent Laboratory of Experimental Dentistry, Medical University of Bialystok, 15-089 Białystok, Poland

**Keywords:** lipid profile, titanium implants, miniplates and screws, dentofacial deformities

## Abstract

**Background/Objectives:** The objective of this study was to analyze the profiles of sphingosine (Sph), sphinganine (SPA), sphingosine-1-phosphate (S1P), and ceramides (C14 Cer, C16 Cer, C18:1 Cer, C18 Cer, C20 Cer, C22 Cer, C24:1 Cer, and C24 Cer), along with caspases (CAS-3, CAS-6, and CAS-9), in serum and in the periosteum of the maxilla and mandible in patients with dentofacial deformities undergoing treatment with titanium fixations (miniplates and miniscrews). **Methods:** The study group comprised 20 patients who underwent bilateral jaw osteotomy due to dentofacial deformities. The osteotomy segments were stabilized with titanium alloy miniplates and screws. The control group consisted of 20 patients who had not yet received surgical treatment for maxillofacial defects. **Results:** Sphinganine (SPA) and ceramide C22 (C22 Cer) were the only compounds found to be significantly elevated in the serum of the study group compared to the control group. The concentrations of Sph, SPA, C14 Cer, C16 Cer, C18 1 Cer, C18 Cer, C22 Cer, C24 1 Cer, C24 Cer, and S1P were significantly lower in the maxillary periosteum of patients in the study group compared to those in the control group. The concentration of C20 Cer was significantly higher in the maxillary periosteum of patients in the study group compared to the control group. In contrast, the concentrations of Sph, SPA, C14 Cer, C16 Cer, C18 1 Cer, C22 Cer, C24 1 Cer, and C24 Cer were significantly lower in the mandibular periosteum of the study group compared to the control group. The concentrations of C20 Cer and S1P were significantly elevated in the mandibular periosteum of patients in the study group compared with the control group. The activity of CAS-3 was significantly higher in the mandibular periosteum of patients in the study group compared to those in the control group. **Conclusions:** Titanium fixations induce local changes in the sphingolipid profile within the periosteum of the maxilla and mandible, while no systemic impact on this metabolism was observed.

## 1. Introduction

Reconstructive, regenerative, and aesthetic medicine are some of the fastest growing clinical areas, attracting attention of researchers. This is due to the rapidly increasing demands for advanced strategies that will not only lead to reconstruction of dysfunctional tissues and organs, but also to restoring their normal functions. In this context, bone regeneration, especially in the case of large skeletal deviations, injuries, or cancer becomes a big challenge.

The standard treatment for dentofacial deformities, injuries, or tumors of the face typically involves surgical intervention using titanium alloy plates and screws.

Despite the manufacturer’s claims of biocompatibility for titanium and its alloys, an increasing number of reports highlight the need for titanium implant removal. This is attributed to the long-term side effects observed by numerous researchers that arise from leaving titanium components at the implantation site [1,2,3,4]. The surface of titanium implants is coated with a passive layer of titanium dioxide (TiO_2_). While this layer is intended to reduce the corrosion potential of the alloy, many patients develop the phenomenon known as metallosis [3,5,6]. This process leads to the corrosion of the alloy and the accumulation of metallic particles at the implantation site. It is important to note that implants subjected to high loads and stresses, such as those in the mandible, are especially prone to metallosis [3,5,6]. The mere presence of a titanium implant, and even more so its wear products, disrupts the body’s immune processes, triggering oxidative stress and inflammation. These reactions not only necessitate further surgeries but may also result in complications affecting distant organs. Borys J. et al. demonstrated that exposure to Ti6Al4V titanium alloy disrupts the redox balance, as well as the levels of cytokines, chemokines, and growth factors in the periosteum surrounding titanium implants in the maxilla and mandible [1]. They also observed that titanium implants placed in the mandible, but not in the maxilla, led to an increase in the production of pro-inflammatory cytokines and induced apoptosis [2].

Studies have shown that redox balance is closely linked to the function of membrane rafts, particularly lipid rafts, which play a key role in initiating and transmitting redox signaling as well as activating antioxidant enzymes [7]. Observations suggest that in the case of titanium-related antioxidant and oxidant disturbances, we can expect deviations in the lipid profile of the periosteum covering the implants. The assessment of the lipid profile seems to be extremely important because lipids play a crucial role in bone remodeling and healing. It has been shown that the presence of lipids in the trabecular area of cortical bone reduces the permeability of radicals, potentially influencing the metabolic functions of osteoblasts and osteocytes [8]. Studies have shown that lipids, particularly sphingolipids, play a crucial role in determining the fate of skeletal progenitor cells and in regulating the key interaction that underpins bone homeostasis, namely, the crosstalk between osteoblasts and osteoclasts. These findings emphasize the critical importance of sphingolipid metabolism in bone tissue remodeling [9]. Furthermore, sphingolipids are a group of biologically active lipids that regulate essential cellular processes, including inflammation, proliferation, differentiation, growth arrest, and apoptosis. It is important to note that ceramide (Cer) and sphingosine-1-phosphate (S1P) have opposing roles in the regulation of these processes. Ceramide is a pro-apoptotic molecule, while S1P promotes proliferation. The ratio between S1P and Cer, often referred to as the sphingolipid rheostat, plays a crucial role in determining whether cells will undergo proliferation or apoptosis [10,11].

The present study aims to evaluate the profiles of key molecules involved in sphingolipid metabolism, including sphingosine (Sph), sphinganine (SPA), sphingosine-1-phosphate (S1P), and various ceramides (C14 Cer, C16 Cer, C18 1 Cer, C18 Cer, C20 Cer, C22 Cer, C24 Cer, and C24 1 Cer) in the maxillary and mandibular periosteum of patients with dentofacial deformities who are undergoing treatment with titanium fixation devices.

## 2. Materials and Methods

### 2.1. Ethical Considerations

Participants from both the study and control groups were fully informed about the nature and objectives of the study, the methods of sample collection (blood and periosteum), and the potential risks of complications. They provided informed, written consent to participate in the research. Approval for the study was granted by the Bioethics Committee of the Medical University of Bialystok (APK.002.71.2023 and APK.002.72.2023).

### 2.2. Patients of Study and Control Groups

The study involved 40 patients (26 women and 14 men) of Polish nationality, who had been diagnosed with class III skeletal defects of the face according to Angle’s classification (characterized by excessive growth of the maxilla and/or hypotrophy of the mandible). These patients were underwent a maxillary and mandibular osteotomy (bimaxillary osteotomy). The osteotomy segments of jaws bone were moved to the planned occlusal and aesthetic position and then immobilized using titanium miniplates and screws.

Both the study and control group participants were treated at the Department of Maxillofacial and Plastic Surgery at the Medical University of Bialystok, Poland, between 19 January 2023 and 30 November 2024. All surgical procedures were carried out by an experienced surgeon (J.B.), a specialist in maxillofacial surgery, along with his team of assistants.

Patients aged 20–30 years, (mean age: 25 years and 7 months) were included in the study and control groups. They were generally healthy, without systemic diseases (autoimmune, cancer, inflammatory, cardiological, metabolic, focal, or mental diseases) or eating disorders such as anorexia or bulimia. The patients did not have any concurrent or previous local diseases (dental pulpitis, periodontitis, oral mucositis, osteomyelitis, or cancer); had not used dental restorations or braces; and had not previously received titanium bone implants, joint prostheses, vascular clamps, orthodontic screws, or dental implants. They were not taking any medications or dietary supplements.

Patients with inflammatory conditions and other diseases, either local or systemic, were excluded from the study and control groups. Patients with poor oral hygiene, cigarette or e-cigarette smokers, and alcohol or drug users were also excluded from both groups.

The study group consisted of 20 patients (12 women and 8 men), aged between 20 and 28 years (average age 24 years and 2 months), who underwent bimaxillary osteotomy to correct dentofacial deformities with the use of titanium fixations. After the osteotomy, fragments were relocated and placed in the planned occlusal position. Then, they were fixated with miniplates and screws constructed from the titanium alloy Ti6Al4V (ChM Lewickie Sp. z o. o., Lewickie, Poland). Four 4-hole miniplates were applied to the maxilla and mandible (two miniplates on the right and left sides), each secured with four screws. The titanium bone fixations were surgically removed under general anesthesia approximately 10 months after the bimaxillary osteotomy procedure, once bone fusion was confirmed. Bone union was evaluated using control radiographs taken routinely before the removal of the titanium miniplates and screws, as well as during the surgery. Radiological diagnostics included a teleroentgenogram in the PA (posterior–anterior) projection and cephalometric imaging in the lateral projection (Cranex 3D, Soredex, PaloDex Group Oy Nahkelantie 160, Finland, FI-04300, Hyrylä, Finland).

The control group included 20 patients (14 women and 6 men), aged between 21 and 30 years (average age 25 years and 3 months), undergoing surgical treatment for dentofacial deformities before osteotomy of jaws and placement titanium fixations.

One month prior to the procedure, immediately after it, and until the removal of the titanium bone fixations, patients followed a balanced diet (2200 kcal: 55% carbohydrates, 30% fat, and 15% protein) created by a dietitian and were kept under his supervision. While at the Department of Maxillofacial and Plastic Surgery, the patients were thoroughly examined both before and after surgery, as well as following their hospital stay. Additionally, they attended follow-up appointments to assess their general and local health condition in detail. No inflammatory, allergic, or neurological complications were observed in the patients selected for the study.

Moreover, during treatment, following the osteotomy of the jaws and the placement of titanium miniplates and screws, no complications related to the presence of titanium fixations in the maxilla and mandible occurred during the healing process of the bones. None of the patients were excluded from the study.

### 2.3. Material Collection

Blood for biochemical tests was collected from the patient in the morning, after a night’s rest, on an empty stomach, while collecting blood for necessary tests routinely performed before surgery. Blood in the amount of 10 mL was collected from the antecubital vein into test tubes (Serum CAT, S-Monovette, Sarstedt, Germany). Serum samples were isolated by centrifugation at 3000 rpm and 4 °C (EBA 200, Hettich Zentrifugen, Tuttlingen, Germany) for 15 min and subsequently stored at −80 °C until further biochemical testing.

In the study group, grayish discolored periosteum from the maxilla (Max Study) and mandible (Man Study) located adjacent to the miniplates and screws (3 mm × 7 mm in size and 1 mm thick) was collected from the same patient during the routine procedure for the removal of titanium bone fixations. Typically, these periosteum samples are discarded after the procedure.

In the control group, small pieces of healthy periosteum (3 mm × 7 mm, 1 mm thick) from both the maxilla (Max Control) and mandible (Man Control) were collected from the same patient during surgery, specifically during the osteotomy of the maxilla and mandible, before the insertion of titanium fixations. After collection, periosteum samples from both the control and study groups were placed in Eppendorf-type tubes (MEDLAB PRODUCTS, Raszyn, Poland). These were 2 mL lens-bottom, graduated tubes with a labeling field made of high-transparency, colorless material and equipped with lids. The precise sealing of the Eppendorf tube caps ensures minimal evaporation during long-term storage. The Easy-Lock lid prevents uncontrolled popping open during incubation. They are suitable for basic applications such as enzymatic degradation, plasmid DNA isolation, and storage of samples and reagents. Samples of maxillary and mandibular periosteum were placed in tubes and stored at −80 °C until biochemical testing.

Sections of periosteum covering the osteotomy segments are typically removed prior to the insertion of titanium miniplates and screws.

### 2.4. Preparation of Periosteum Homogenates

The periosteum samples (20 mg) from the maxilla and mandible were homogenized with the Bead Ruptor Elite (Omni International, Kennesaw, GA, USA) in a buffer solution composed of 0.25 M sucrose, 25 mM KCl, 50 mM Tris, and 0.5 mM EDTA, at pH 7.4.

### 2.5. Measurement of Sphingolipids

Sphingolipid levels were quantified using ultra-high-performance liquid chromatography coupled with tandem mass spectrometry (UHPLC/MS/MS) following the method described by Blachnio-Zabielska et al., with slight modifications [12]. For each sample (tissue homogenates or plasma), 50 μL of an internal standard mix (ISTD), which included Sph-d7, SPA-d7, S1P-d7, C15:0-d7-Cer, C16:0-d7-Cer, C18:1-d7-Cer, C18:0-d7-Cer, 17C20:0-Cer, C24:1-d7-Cer, and C24-d7-Cer (Avanti Polar Lipids, Alabaster, AL), and 2 mL of an extraction solution (isopropanol:water:ethyl acetate, 30:10:60; *v*/*v*/*v*) were added. The mixture was vortexed, sonicated, and centrifuged at 4000 rpm at 4 °C for 10 min (Sorvall Legend RT). The supernatants were collected into new vials, and the remaining pellets were re-extracted. The combined supernatants were evaporated under nitrogen, and the dried residues were reconstituted in LC Solvent B (2 mM ammonium formate, 0.1% formic acid in methanol) for UHPLC/MS/MS analysis.

Sphingolipids, including sphingosine (Sph), sphingosine-1-phosphate (S1P), sphinganine (SPA), and various ceramides (C14:0-Cer, C16:0-Cer, C18:1-Cer, C18:0-Cer, C20:0-Cer, C22:0-Cer, C24:1-Cer, and C24:0-Cer), were separated on a reverse-phase column (Zorbax SB-C8, 2.1 × 150 mm, 1.8 μm) using a binary gradient (Solvent A: 1 mM ammonium formate, 0.1% formic acid in water; Solvent B: 2 mM ammonium formate, 0.1% formic acid in methanol) at a flow rate of 0.4 mL/min. Quantification was performed using a Sciex Qtrap 6500+ mass spectrometer (SCIEX, Framingham, MA, USA) with positive electrospray ionization (ESI), except for S1P, which was analyzed in negative mode, utilizing multiple reaction monitoring (MRM) against standard curves for each analyte.

The sphingolipid rheostat was calculated as the ratio of S1P concentration [pmol/mL] to total ceramide [pmol/mL] in serum or S1P [pmol/mg tissue] to total ceramide [pmol/mg tissue] in maxillary or mandibular periosteum [10,13].

### 2.6. Measurement of Caspases

Caspase-3 (CAS-3, EC 3.4.22.56) activity was assessed using a colorimetric method, with Ac-Asp-Glu-Val-Asp-pNA as the substrate. The absorbance was measured at 405 nm [14]. Caspase-9 activity (CAS-9, EC 3.4.22.62) was quantified using Ac-Leu-Glu-His-Asp-pNA as the substrate. Caspase-6 activity (CAS-6, EC 3.4.22.59) was determined fluorometrically with Z-VEID-AFC as the substrate [15].

### 2.7. Statistical Analysis

The analysis of the data was performed using GraphPad Prism 10.2.0 statistical software for macOS (GraphPad Software, La Jolla, CA, USA). The normality of the data distribution was evaluated using the Shapiro–Wilk test. Due to a lack of normal data distribution, group comparisons were conducted using the Mann–Whitney U-test. The results were visualized with violin plots, displaying the median, quartiles, and the range (minimum to maximum). A *p*-value of less than 0.05 was considered statistically significant.

The sample size was determined based on the earlier conducted pilot study, with the statistical power of the test set at 0.9.

## 3. Results

### 3.1. Serum

**Figure 1:**
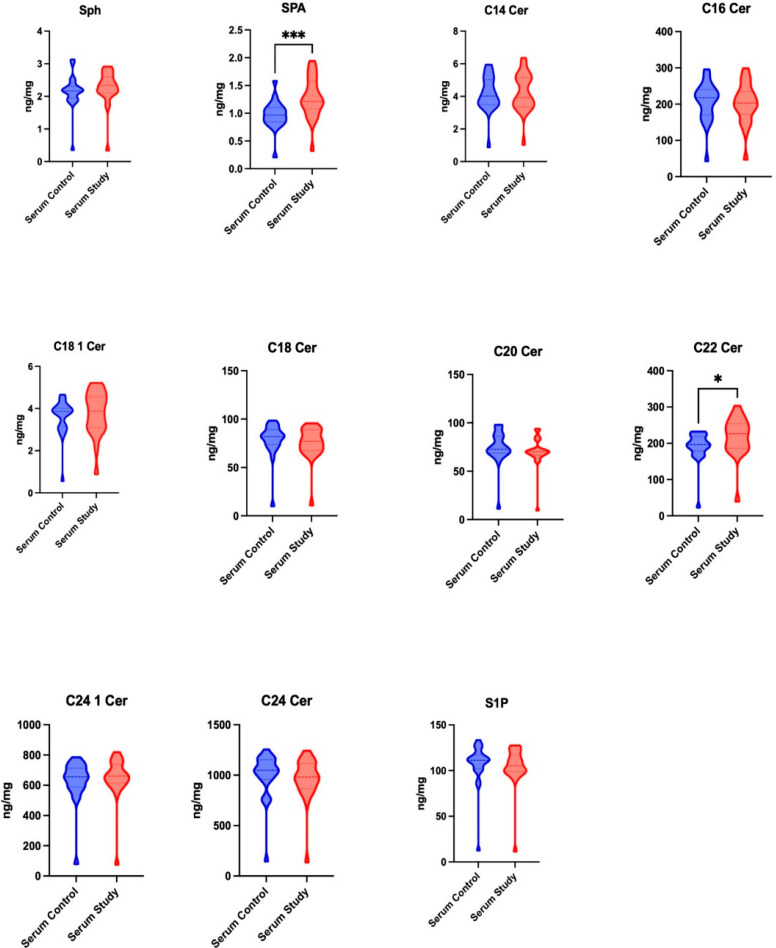
The concentrations of sphingolipids, including sphingosine (Sph), sphinganine (SPA), and sphingosine-1-phosphate (S1P), and ceramides, including ceramide C14 (C14 Cer), ceramide C16 (C16 Cer), ceramide C18 1 (C18 1 Cer), ceramide C18 (C18 Cer), ceramide C20 (C20 Cer), ceramide C22 (C22 Cer), ceramide C24 1 (C24 1 Cer), and ceramide C24 (C24 Cer), in the serum of patients in both the control and study groups. * *p* < 0.05, *** *p* < 0.0005.

Except for SPA and C22 0 Cer, which showed significantly higher concentrations in the serum of the study group compared to the control group (*p* = 0.0007 and *p* = 0.0239, respectively), no significant differences were observed in the other parameters tested (Figure 1).

### 3.2. Maxillary Periosteum

**Figure 2:**
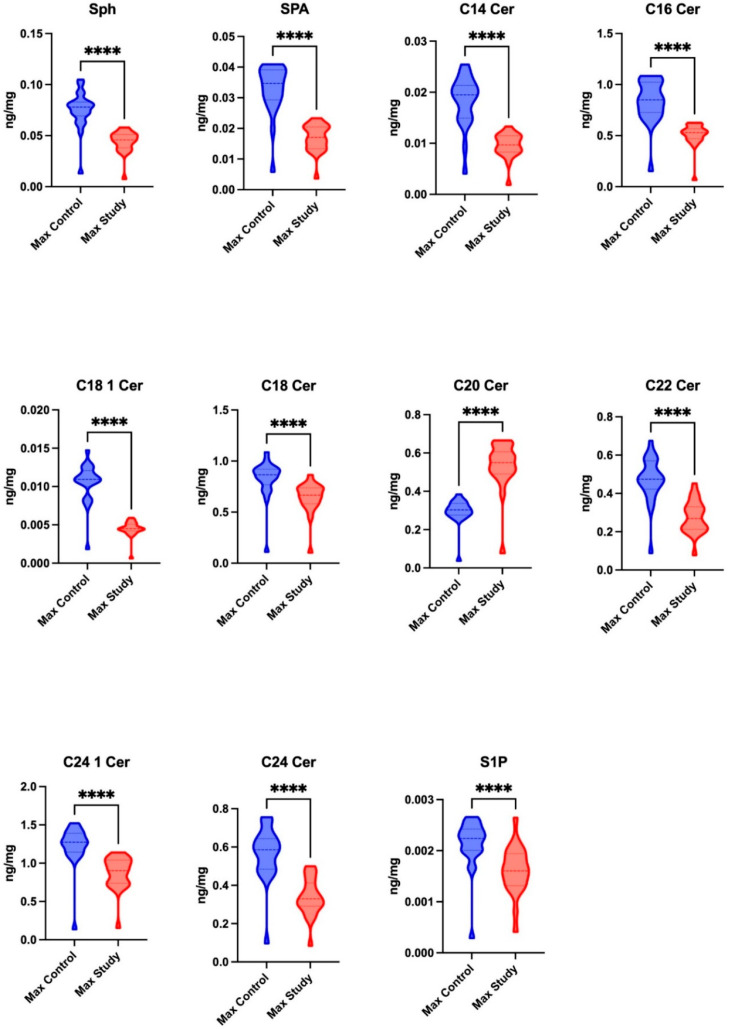
The concentrations of sphingolipids, including sphingosine (Sph), sphinganine (SPA), and sphingosine-1-phosphate (S1P), and ceramides, including ceramide C14 (C14 Cer), ceramide C16 (C16 Cer), ceramide C18 1 (C18 1 Cer), ceramide C18 (C18 Cer), ceramide C20 (C20 Cer), ceramide C22 (C22 Cer), ceramide C24 1 (C24 1 Cer), and ceramide C24 (C24 Cer), in the maxillary periosteum of the patients in the control and study groups. **** *p* < 0.0001.

The study group exhibited significantly reduced concentrations of Sph (*p* < 0.0001), SPA (*p* < 0.0001), C14 Cer (*p* < 0.0001), C16 Cer (*p* < 0.0001), C18 1 Cer (*p* < 0.0001), C18 Cer (*p* < 0.0001), C22 Cer (*p* < 0.0001), C24 1 Cer (*p* < 0.0001), and C24 Cer (*p* < 0.0001) in the maxillary periosteum compared to the control group. Conversely, the levels of C20 Cer (*p* < 0.0001) and S1P (*p* < 0.0001) were significantly higher in the study group relative to the control group (Figure 2).

**Figure 3:**
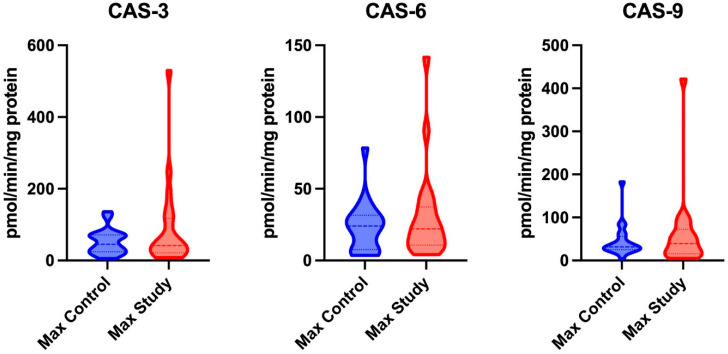
The activity of Caspase-3 (CAS-3), Caspase-6 (CAS-6), and Caspase-9 (CAS-9) in the maxillary periosteum of patients from both the control and study groups.

The activity levels of CAS-3, CAS-6, and CAS-9 in the maxillary periosteum showed no significant differences between the control and study groups (Figure 3).

### 3.3. Mandible Periosteum

**Figure 4:**
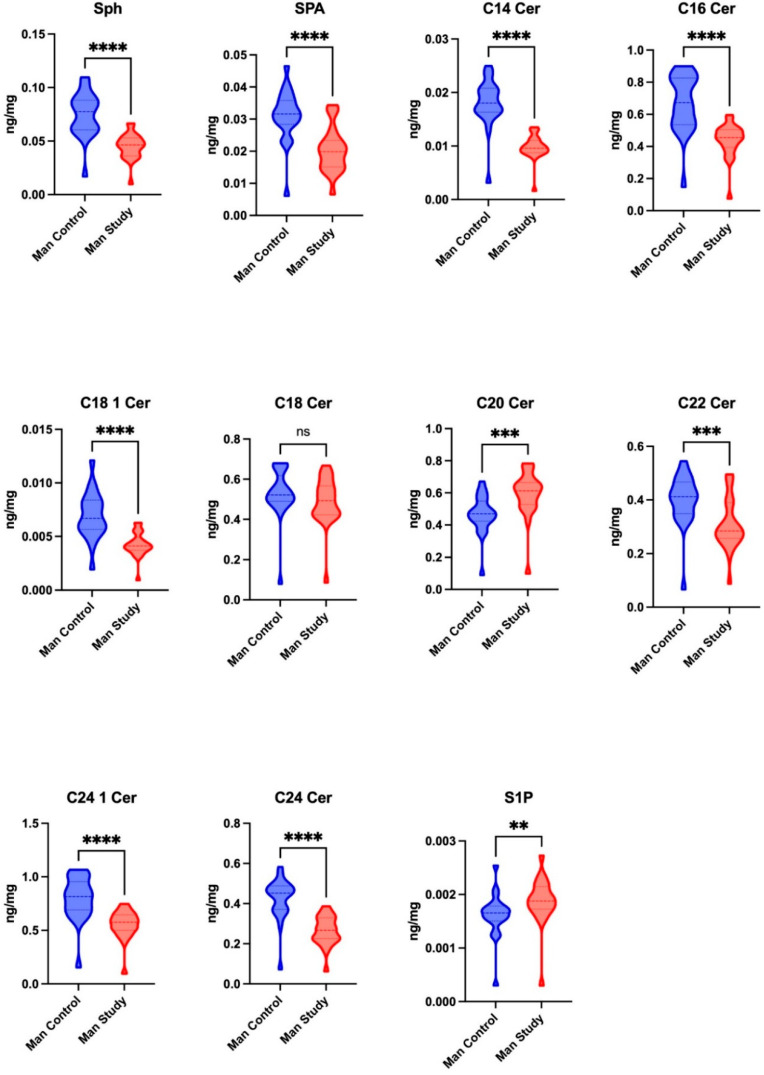
The concentrations of sphingolipids, including sphingosine (Sph), sphinganine (SPA), and sphingosine-1-phosphate (S1P), and ceramides, including ceramide C14 (C14 Cer), ceramide C16 (C16 Cer), ceramide C18 1 (C18 1 Cer), ceramide C18 (C18 Cer), ceramide C20 (C20 Cer), ceramide C22 (C22 Cer), ceramide C24 1 (C24 1 Cer), and ceramide C24 (C24 Cer), in the mandibular periosteum of the patients in the control and study groups. ** *p* < 0.005, *** *p* < 0.0005, **** *p* < 0.0001, ns—not significant.

The concentrations of Sph (*p* = 0.0046), SPA (*p* < 0.0001), C14 Cer (*p* < 0.0001), C16 Cer (*p* < 0.0001), C18 1 Cer (*p* < 0.0001), C22 Cer (*p* = 0.0005), C24 1 Cer (*p* < 0.0001), and C24 Cer (*p* < 0.0001) were significantly reduced in the mandibular periosteum of patients in the study group compared to those in the control group. The concentrations of C20 Cer and S1P were significantly higher in the mandibular periosteum of patients in the study group (*p* = 0.0002) compared to those in the control group (*p* < 0.005), while no significant difference was observed in the concentrations of C18 Cer between the two groups (Figure 4).

**Figure 5:**
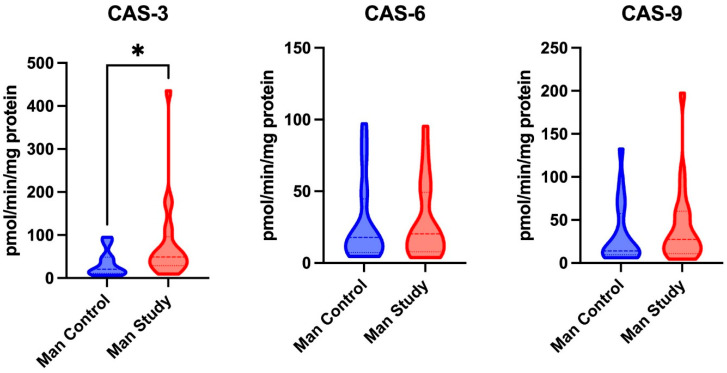
The activity of Caspase-3 (CAS-3), Caspase-6 (CAS-6), and Caspase-9 (CAS-9) in the mandibular periosteum of patients from both the control and study groups. * *p* < 0.05.

CAS-3 activity was significantly higher in the mandibular periosteum of patients in the study group (*p* = 0.0154) compared to those in the control group. No significant differences were found in the activity of CAS-6 and CAS-9 in the mandibular periosteum between the study and control groups (Figure 5).

### 3.4. Sphingolipid Rheostat

The sphingolipid rheostat in the mandibular periosteum was significantly higher in the study group compared to the control group (*p* < 0.0001) (Table 1).

Rheostat values are presented as the ratio of S1P [pmol/mL] to total ceramide [pmol/mL] in serum or as the ratio of S1P [pmol/mg tissue] to total ceramide [pmol/mg tissue] in the maxillary periosteum (Max) or mandibular periosteum (Man).

## 4. Discussion

Permanent or temporary metal implants, mainly Ti6Al4V titanium alloy implants, like titanium plates and screws, are commonly used in maxillofacial and orthopedic surgery. Such implants are believed to exhibit a high degree of biocompatibility, although there are numerous reports suggesting that they should be removed after a period of osteointegration [1,3,16,17]. Disruptions caused by titanium in tissues can include various physiological and biochemical changes. The accumulation of titanium particles in surrounding tissues, such as the periosteum, can lead to metallosis, where the body reacts to the metal as a foreign substance. This can trigger inflammatory responses and disrupt normal tissue function [2,17,18,19,20,21]. On a local level, the presence of titanium can impair antioxidant defense mechanisms, leading to oxidative and nitrosative stress. These stresses occur when there is an imbalance between the production of reactive oxygen and nitrogen species and the body’s ability to neutralize them, potentially damaging cellular structures [1,2,18]. Additionally, disturbances in glutathione metabolism, an essential antioxidant, and mitochondrial function may occur, further compromising the tissue’s ability to repair and regenerate [17]. These disruptions are often associated with chronic inflammation, tissue degeneration, and, in some cases, the failure of the implant itself [18,19,20,21].

Titanium implants can also be a source of metallic particles detected in the blood and distant organs such as lymph nodes, lungs, liver, spleen, and kidneys. Titanium particles can influence the development of dermatological diseases and neurological disorders, such as Parkinson’s disease and Alzheimer’s disease [21]. Titanium may lead to a systemic immune inflammatory response due to the activation of the complement system, neutrophils, lymphocytes, and macrophages [18,20,21]. Titanium particles can activate macrophages, leading them to release pro-inflammatory cytokines and chemokines. Additionally, titanium may influence macrophage polarization. Macrophages can shift between different activation states, such as the pro-inflammatory M1 phenotype and the anti-inflammatory M2 phenotype. If macrophages remain in the M1 state for an extended period, titanium particles may promote a more chronic, inflammatory environment [22].

The process of bone tissue regeneration involves a variety of highly organized physiological processes, including bone formation and resorption [23]. As has been shown, sphingolipids are bioactive factors that control intracellular membrane function. Their presence in the porous structure of cortical bone limits radial permeability, thereby regulating the metabolic activity of osteoblasts and osteocytes [8]. Moreover, the optimal sphingolipid content in bone cell membranes determines the development and differentiation of skeletal progenitor cells [8]. Lipids are essential in regulating signaling pathways and cellular function in bone biology, which suggests their relevance to pathological processes. Indeed, sphingolipid metabolism has been shown to play a key role in osteogenesis. Recent research highlights the complex effects of sphingolipids on osteoblasts, osteoclasts, and the crucial interactions that maintain bone homeostasis, specifically, the balance between osteoblast and osteoclast activity. It also emphasizes the multifaceted role of sphingolipid metabolism in bone remodeling [9].

The present study is the only one to estimate the content of selected lipid groups, including sphingosine (Sph), sphinganine (SPA), sphingosine-1-phosphate (S1P), and ceramides (C14 Cer, C16 Cer, C18 1 Cer, C18 Cer, C20 Cer, C22 Cer, C24 1 Cer, and C24 Cer), in the periosteum of the maxilla and mandible undergoing osteointegration with Ti4Al4V titanium implants. In summary, the concentration of the majority of the tested parameters was significantly reduced in the periosteum of the maxilla and mandible in the study group when compared to the control group. The concentrations of C20 Cer and S1P were the only ones significantly higher in the periosteum of the maxilla and mandible in the study group compared to the control group. When analyzing the sphingolipid rheostat, a significant increase was found only in the periosteum of the mandible in the study group, as compared to the control group. The periosteum was collected after a period of 10 months, i.e., when osteointegration was clinically and radiologically confirmed. The collected periosteum had a gray coloration, which our previous studies had shown to be the result of the deposition of ions contained in the titanium implants and screws. Energy-dispersive X-ray spectroscopy (EDS) showed significantly higher levels of titanium, aluminum, and vanadium. These elements were undetectable in the periosteum of the control group [2,17].

Our study showed only slight differences in the evaluated parameters in the serum of the studied patients (↑SPA, ↑C22 Cer vs. control), suggesting a local effect of titanium implants on the metabolism of the studied group of compounds. We obtained similar results by evaluating the antioxidant barrier and oxidative stress markers in the blood serum and blood cells of patients undergoing osteointegration of maxillary and mandibular bones due to dentofacial deformities [1]. We also did not observe any correlations between the results of the evaluated parameters in the blood and those obtained in the maxillary and mandibular periosteum. This result again suggests no systemic effect of titanium strains on the content of the selected lipid groups in the blood.

Abnormal ceramide content can cause osteoblast disorders and dysfunction and disrupt bone remodeling processes, so our results may have clinical relevance. In patients undergoing osteosynthesis with titanium fixations, the concentrations of Sph, SPA, C14 Cer, C16 Cer, C18 1 Cer, C22 Cer, C24 1 Cer, and C24 Cer in the periosteum of both the maxilla and mandible were notably lower compared to those in the control group [24]. In the study group, the concentrations of C20 Cer and SP1 were elevated in the mandibular periosteum, while C20 Cer alone was significantly higher in the maxillary periosteum, compared to the control group.

This increase raises some concerns, as studies by Beom-Jun Kim et al. have shown that elevated ceramide levels may contribute to an increased susceptibility of bones to fractures in the future [25]. This fact is due to an increase in osteoclast differentiation and their activity. 

A rise in ceramide concentration appears to be responsible for inducing apoptosis in osteoblasts and chondrocytes and also increasing osteoclast survival. Deficiency, or a reduction in ceramide concentrations, can cause inhibition of apoptosis and further consequences, as we discuss below [25]. Thus, it would seem that with changes in the content of the studied ceramides, we should observe changes in the parameters of enzymes responsible for apoptosis. However, our study showed that the activities of pro-apoptotic enzymes CAS-3, CAS-6, and CAS-9 in the periosteum of the maxilla and the activity of CAS-6 and CAS-9 in the mandibular periosteum of the patients in the study group showed no difference compared to the control group. In the mandibular periosteum covering titanium fixations (miniplates and screws), we noted an increase in CAS-3 activity, similar to our previous studies, where we evaluated the periosteum of mandibular trauma patients undergoing plate osteosynthesis [2]. Studies have shown that processes accompanying bone healing after trauma are largely determined by the clinical picture of the patient, especially the clinical situation requiring the fusion of bone fragments [23]. The study group of the described experiment consisted of patients with deformations of the maxilla and mandible, while the study group in the previous experiment were patients with different double-sided fractures (beating, engaging in sports, car accidents, or unfortunate falls) [2]. It also appears that the duration of exposure to titanium fixations that remain in the bone may also be of significance. As research has shown, the effect of titanium on surrounding cells is greatest in the initial period, decreasing over time, which perhaps relates to the phenomenon of apoptosis [26]. In vitro studies have shown that titanium particles can induce osteoblast apoptosis, which in turn inhibits bone formation around implants [27]. The findings of this study suggest that both the amount of wear debris generated and its local accumulation may play a crucial role in influencing bone cell function [27]. Thus, in the mandible, which is a mobile bone, the tribological wear processes of titanium fixations (fretting corrosion) and the release of titanium particles may be more pronounced than in the maxilla, so perhaps some proapoptotic parameters may be elevated in the mandibular periosteum. However, long-term monitoring of the influence of titanium particles on tissues is recommended, as some studies suggest that they may impact the genomic instability of structural cells of connective tissue [16].

It is well documented that S1P and ceramides maintain the so-called rheostat, which provides a balance between proliferation and cell survival phenomena associated with apoptosis [28]. As previously mentioned, S1P promotes cell proliferation, while ceramides are recognized for their role in inducing apoptosis [28]. The balance between these two signaling molecules is known as the sphingolipid rheostat. Ceramides and S1P are believed to be in negative coupling. Specifically, when ceramide levels decrease, S1P content increases, and vice versa [29]. Evaluating the concentration of S1P, we noted a significant reduction in the level of this molecule in the periosteum of the maxilla and an increase in its concentration in the periosteum of the mandible. These results indicate that after a 10-month exposure to titanium implants and screws, the sphingolipid rheostat in the maxillary and mandibular periosteum is impaired. The cause of this disruption is unclear, although it is known that the rheostat can be disturbed by targeting enzymes that have a direct or indirect impact on this balance.

Ceramide formation is initiated by the activity of various enzymes that are part of independent pathways: de novo ceramide synthesis, the sphingomyelin pathway, and ceramide salvage pathway [29]. It is important to emphasize that hydrolysis of ceramide with formation of sphingosine, followed by its phosphorylation by sphingosine kinase (SPHK1) to S1P, could heavily influence the rheostat. It can therefore be suspected that exposure to titanium implants disrupts one of the ceramide formation pathways, leading to a reduction in ceramide formation, resulting in decreased S1P production.

In the periosteum of the maxilla, we found a reduced sphingolipid rheostat in relation to the control group, which may indicate a reduction in proliferative processes.

The observed increase in the sphingolipid rheostat in the mandibular periosteum of the study group suggests a localized alteration in sphingolipid metabolism in response to titanium plate fixation. It is well known that S1P promotes cell survival, proliferation, and angiogenesis, whereas ceramides are associated with pro-apoptotic and anti-proliferative effects. The elevated sphingolipid rheostat indicates a relative increase in S1P signaling, which may contribute to enhanced osteogenesis, tissue remodeling, or inflammatory responses specifically in the mandibular region. From a clinical perspective, this shift in the sphingolipid rheostat may influence the dynamics of bone healing and physiological remodeling processes. Estimating the rheostat in the mandibular periosteum and observing a significantly higher ratio of S1P to total Cer compared to the control group can indicate that in the periosteum of the mandible, proliferative processes prevail over apoptotic ones. Is this a favorable outcome? Not entirely, as it is known that inhibition of apoptosis with increased proliferation is one of the characteristics of cancer cells [29]. The research conducted by Coen et al. explored the effects of particulate titanium debris from a metal prosthesis on the induction of genomic instability and tetraploidy. The genomic instability assessed in this study included lethal mutations, delayed reproductive death, and delayed cytogenetic abnormalities in the progeny cells exposed to the debris [16]. This finding may indicate the necessity for long-term monitoring of patients following procedures involving titanium implants and clearly highlights the importance of removing them after the union with bone.

With regard to dental implants, which can also be made of titanium alloys, they are left in the bone for many years, sometimes for the patient’s entire lifetime. From the moment a dental implant is introduced into human tissues, it remains a functional implant and plays a crucial role in both aesthetics and function, contributing to the rehabilitation of the masticatory system and ensuring patient comfort. It represents a modern solution for the treatment of tooth loss [1,30].

Miniplates and screws used for maxillary bone fixation are placed on the bone surface and are only necessary during the bone healing period (after fractures, osteotomies, or reconstructions). Once bone union is achieved, they become non-functional implants; however, their presence in human tissues may lead to various clinical issues [1,2,3]. Patients with miniplates and screws sometimes report excessive sensitivity to cold, hyperesthesia, recurrent swelling, palpable irregularities on the bone surface, and even facial deformities [1]. In some patients, allergic reactions may occur [1,18]. Plates placed on the bone surface under a thin layer of oral tissues may hinder the use of removable dentures or orthodontic treatment with Bollard plates. Additionally, screws inserted perpendicularly to the bone surface may significantly complicate or even prevent implant-prosthetic treatment [1]. These fixations may also cause artifacts that interfere with the proper interpretation of CT and MRI scans [1]. We have observed patients in whom, even many years after osteosynthesis, extraoral fistulas developed due to a reaction to the retained fixations, leading to unsightly scarring of the facial and/or neck skin [1]. Our own research, as well as studies conducted by other authors, has demonstrated oxidative stress, chronic inflammation, mitochondrial dysfunction, and metallosis in the tissues surrounding miniplates and screws [1,2,3]. In children, titanium fixations in the jaws may cause growth disturbances, making their removal necessary once clinical bone union is achieved. All of the above data suggest the justification for removing titanium bone fixation issues [1,2]. The surface of these implants is coated with a TiO_2_ layer, ensuring biocompatibility with human tissues, and its structure does not promote bone integration, making removal fixations easier. This allows for the elimination of non-functional titanium implants that have already fulfilled their purpose [1,2].

**The strength of our study** is the choice of a topic that is extremely relevant with an innovative approach, a clearly developed methodology, and results based on appropriate statistical analysis. The study group is carefully selected and standardized in terms of age, type of surgery, and the number of titanium fixations used. The inclusion and exclusion criteria for both the study and the control group have been thoroughly developed.

**The limitation of this work** is that it only evaluates the main representatives of sphingolipid metabolism, which prevents a complete characterization of this process and its disturbances.

**Further studies** are required to determine whether targeted modulation of the sphingolipid rheostat could optimize bone healing or remodeling processes while minimizing potential complications in maxillofacial surgery involving implants, plates, and titanium screws. When assessing the impact of titanium fixations on the surrounding tissues in terms of sphingolipid metabolism, its parameters should be evaluated not only in blood serum and periosteum but also in bone.

## 5. Conclusions

In our study, after 10 months of titanium implant exposure, we observed changes in the examined parameters. The sphingolipid profile in the maxillary and mandibular periosteum differs from that of the control group, but no clinical symptoms of these changes were observed locally. Titanium implants used to stabilize the maxillary and mandibular bones show no systemic effects on sphingolipid metabolism.

The authors currently recommend the use of titanium plates and screws for osteosynthesis. However, based on clinical observations and their own research, considering the numerous adverse clinical situations associated with the presence of titanium bone fixations, their removal is recommended after achieving clinical bone union. It would be advisable to develop new bone fixation systems based on biodegradable materials, such as magnesium alloys, which would be free from the disadvantages of titanium alloy fixations.

## Figures and Tables

**Table 1:** Sphingolipid rheostat for S1P vs. total ceramides [12,15].

	Serum			Max			Man		
	Control	Study	*p*	Control	Study	*p*	Control	Study	*p*
Minimum	0.0386	0.03994	0.7381	0.00037	0.00024	0.9893	0.00034	0.00051	*p* < 0.0001
25% Percentile	0.04593	0.04235		0.00045	0.00043		0.00043	0.00061	
Median	0.04837	0.04971		0.00048	0.0005		0.00049	0.0007	
75% Percentile	0.05201	0.05213		0.00057	0.0006		0.00054	0.00078	
Maximum	0.06039	0.05602		0.00068	0.0008		0.00086	0.00102	

## Data Availability

The authors will make the raw data supporting the conclusions of this article available upon request, without any unnecessary limitations.
